# A Rare Case of Renal Angiomyolipoma and Polycystic Kidney Disease in a Patient with Tuberous Sclerosis

**DOI:** 10.7759/cureus.63031

**Published:** 2024-06-24

**Authors:** Rohan Shah, Inuganti Venkata Renuka, Tejasri Gundapaneni, Haritha Shah

**Affiliations:** 1 Pathology, NRI Medical College, Guntur, IND

**Keywords:** kidney disease, multicystic renal mass, s: tuberous sclerosis, polycystic kidney disease (pkd), renal angiomyolipoma

## Abstract

Renal angiomyolipoma (AML) is a rare benign tumor that follows an autosomal dominant inheritance pattern. Its association with polycystic kidney disease is uncommon, with only a handful of cases documented in the literature. The growth of lesions to a significant size may lead to life-threatening complications. We report a case of a 32-year-old female who presented with a palpable mass and bilateral flank pain. Following clinical assessment and CT examination, the patient underwent a left radical nephrectomy. The resected mass measured 9.3 x 8.2 x 7.5 cm, and the subsequent histopathological examination confirmed the diagnosis as renal AML.

## Introduction

Renal angiomyolipoma (AML) belongs to the category of perivascular epithelioid cellular differentiation tumors (PEComas), primarily recognized as a benign neoplasm [1,2]. These tumors are characterized by the presence of thick-walled blood vessels, smooth muscle, and mature adipose tissue. They may occur sporadically in 80% of cases or as a part of the tuberous sclerosis complex (TSC) in 20% of cases [2,3]. TSC is a rare genetic disorder caused by mutations in TSC1 or TSC2 [3]. These mutations impact mTOR and hence patients may be responsive to specific mTOR inhibitors [3]. TSC often coexists with renal AML, which typically exhibits increased size and a higher risk of hemorrhage when associated with TSC [3]. However, its association with polycystic kidney disease is sparsely documented. Clinical presentation correlates with the lesion's size, with the most severe complication being retroperitoneal hemorrhage due to microvasculature rupture, potentially leading to shock, known as Wunderlich syndrome [2,3,4]. Predisposed individuals include those with a family history of TSC, with females at a slightly higher risk [1,3,5]. In this report, we present the clinical course, diagnosis, and treatment of a case of sporadic AML in a patient with polycystic kidney disease and tuberous sclerosis.

## Case presentation

A 32-year-old female of Indian origin presented to a tertiary care hospital with a complaint of recurring dull aching pain in both flank regions over the past two months. She did not report any other urinary or gastrointestinal complaints but had a medical history of autosomal dominant polycystic kidney disease (ADPKD) and TSC. She had not been taking any over-the-counter medication before she visited the outpatient department. 

During the clinical examination, no significant abnormalities were detected. However, laboratory results indicated severe anemia, which was addressed through blood transfusions. The serum levels of urea and creatinine were elevated, while all other parameters remained within the normal range (Table 1). Urine analysis showed no presence of hematuria or proteinuria, and there was no evidence indicating bacterial growth. Ultrasound (USG) imaging revealed bilaterally enlarged kidneys with multiple anechoic cysts replacing the renal parenchyma. Additionally, an ill-defined, vascularized, hyperechoic lesion measuring 10x8 cm was noted in the left kidney, indicating AML with possible hemorrhage. This finding was confirmed by a CT scan of the kidneys, ureters, and bladder (KUB), which aligned with the ultrasound diagnosis of AML with hemorrhage.

**Table 1:** Laboratory investigations

Parameter	Value	Reference range
Hemoglobin	7 gm/dL	12.0 - 15.0 gm/dL
Red blood cell count	2.4 million/cumm	3.7 - 4.8 million/cumm
Erythrocyte sedimentation rate (ESR)	40 mm/hour	0 - 20 mm/hour
Serum creatinine	6.8 mg/dL	0.52 - 1.04 mg/dL
Serum urea	81 mg/dL	14 - 34 mg/dL

**Figure 1:**
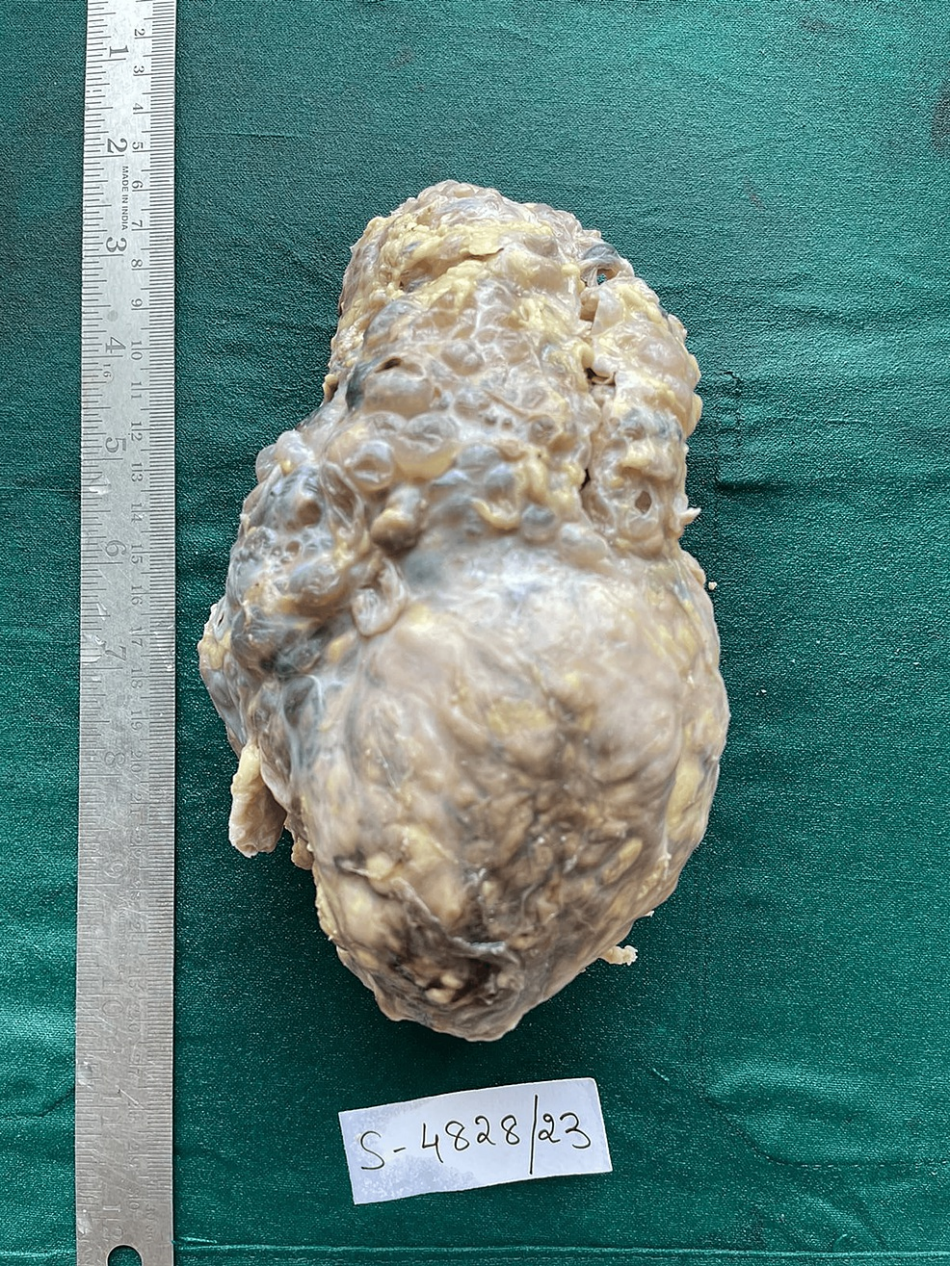
Gross picture of left nephrectomy specimen Nodular enlarged kidney with numerous cysts, varying in size from 0.2 x 0.2 cm to 0.5 x 0.5 cm

**Figure 2:**
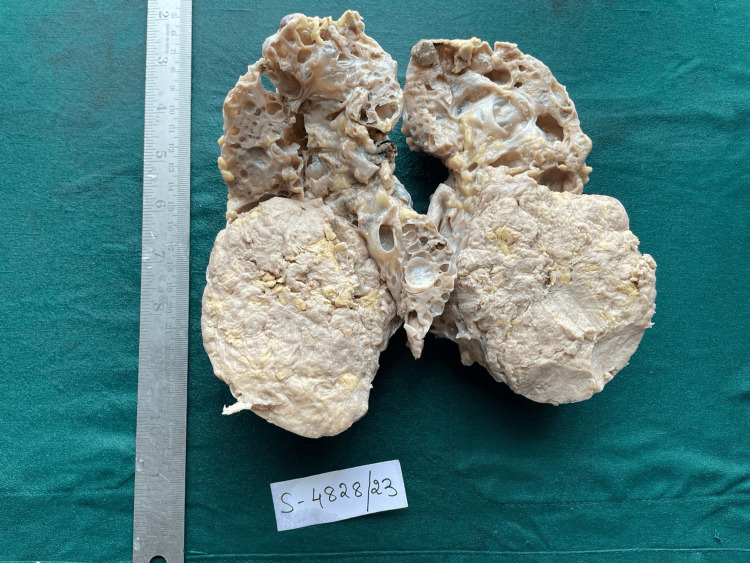
Cut section of left nephrectomy specimen A well-defined, encapsulated, gray-white tumor measuring 9.5 x 8 x 7 cm in the lower pole

**Figure 3:**
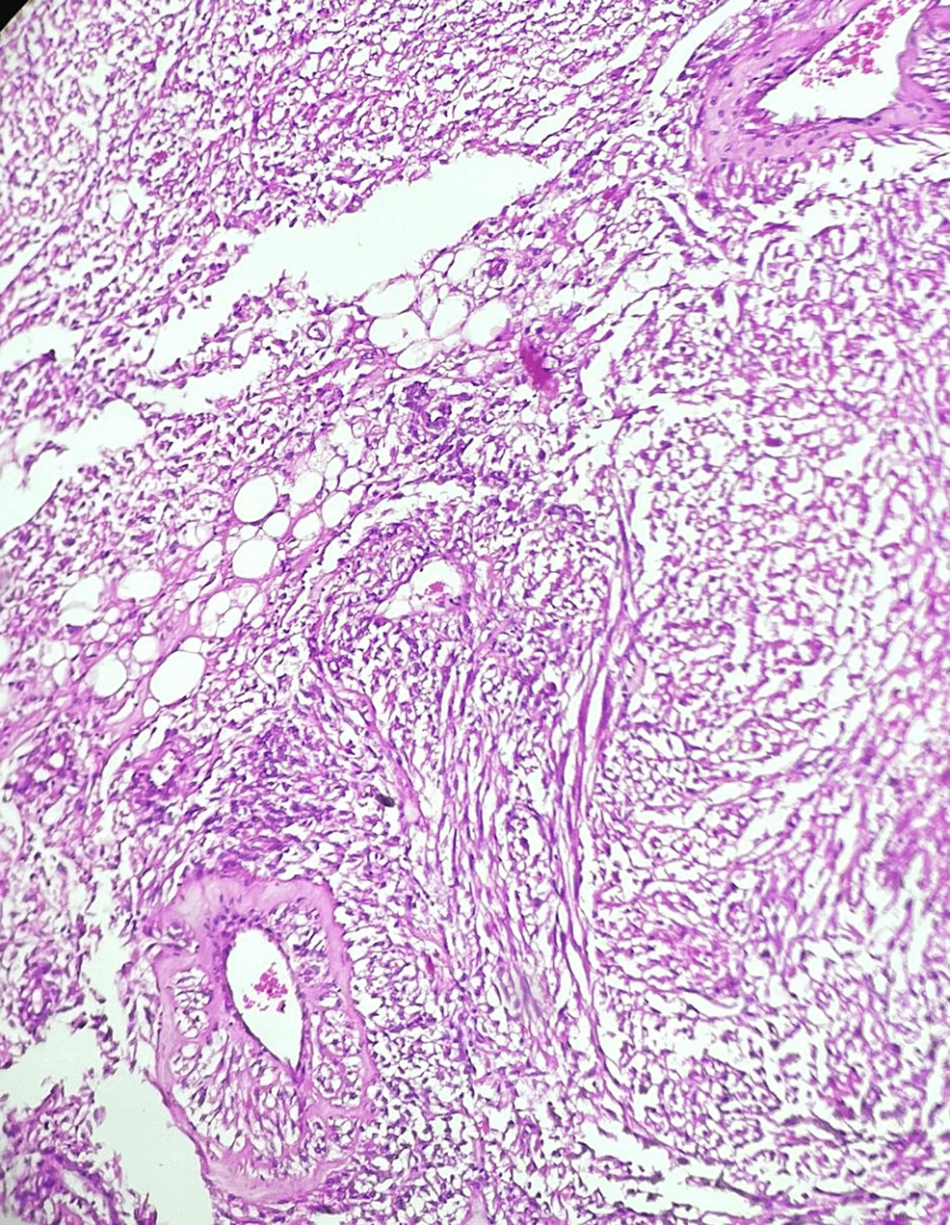
Microphotograph of angiomyolipoma - image 1 Blood vessels with thickened walls, adipose tissue, and smooth muscle bundles (H&E x100)

**Figure 4:**
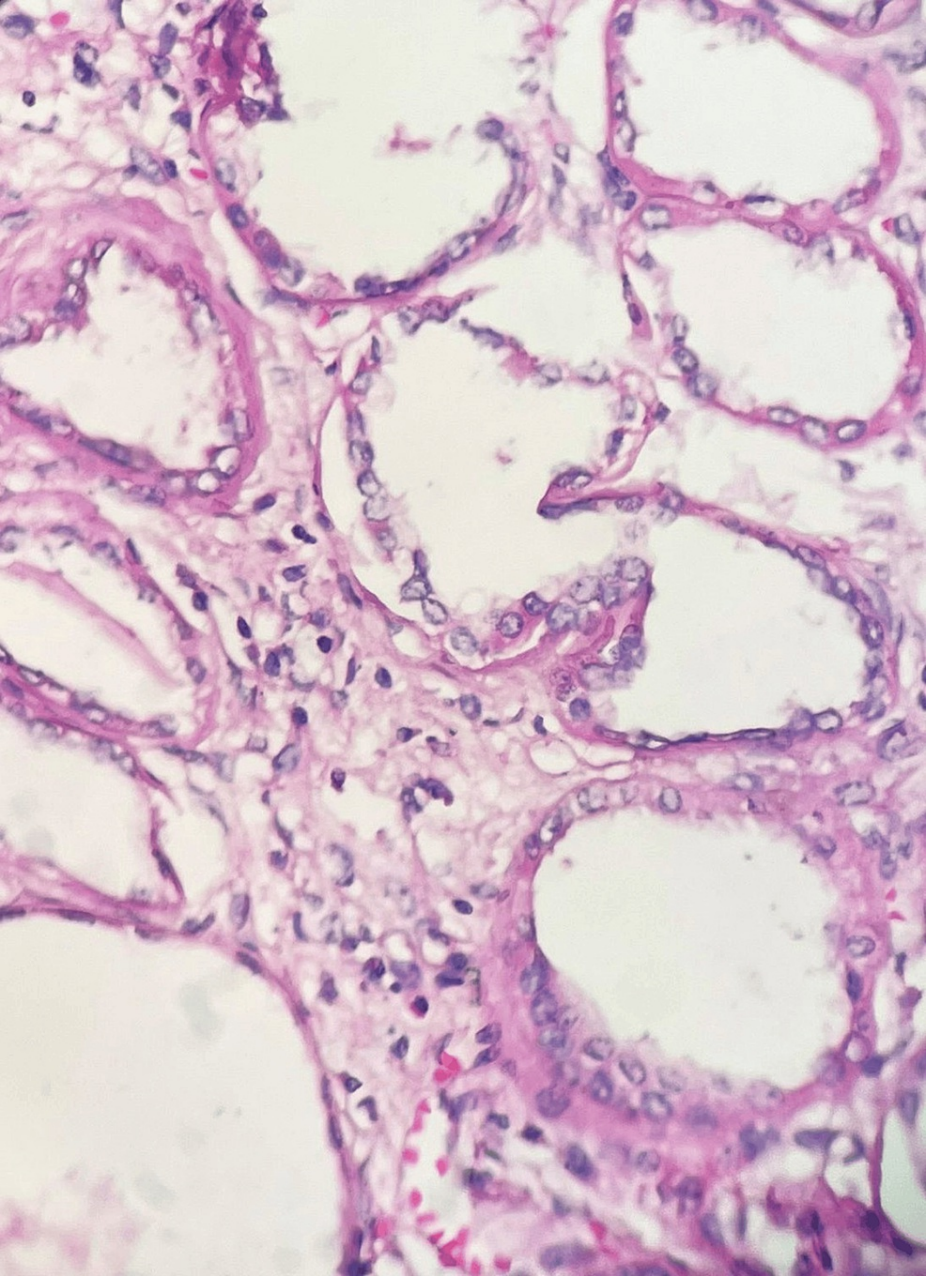
Microphotograph of angiomyolipoma - image 2 Tumor showing thickened blood vessels and cystic spaces (H&E x400)

A left radical nephrectomy was performed, and the specimen was sent for histopathological analysis. The examination revealed a nodular enlarged kidney with numerous cysts, varying in size from 0.2 x 0.2 cm to 0.5 x 0.5 cm (Figure 1). The cut section showed a well-defined, encapsulated, gray-white tumor measuring 9.5 x 8 x 7 cm in the lower pole (Figure 2). Microscopically, the specimen revealed blood vessels with thickened walls, adipose tissue, smooth muscle bundles, and cystic spaces (Figures 3-4).

## Discussion

Renal AML, a rare neoplasm, is commonly referred to as a hamartoma due to its diverse composition, which includes thickened blood vessels, smooth muscle cells, and mature adipose cells, as observed in our case [4]. It manifests in two main variants: typical and atypical [2]. The atypical variant is characterized by increased epithelial cells and decreased adipose tissue, resembling renal cell carcinoma presentation [2]. However, in our case, the presentation aligned with the typical variant, encompassing all three characteristic components. These tumors may arise in association with TSC or sporadically [2,3]. In our case, it occurred in association with both TSC and ADPKD. While AML is not typically associated with renal failure, its co-occurrence with ADPKD increases the risk of severely impaired renal function [6].

Our case manifested a rare combination of both AML and ADPKD, increasing the severity of the condition. Renal AML often presents asymptomatically; however, its association with TSC or other renal conditions can trigger corresponding symptoms, as seen in our case. Symptoms are dependent on size, such as flank pain, palpable abdominal mass, and compression symptoms [5,8]. Our patient, who had preexisting ADPKD and TSC, presented with bilateral flank pain for two months. While postoperative nephrectomy specimens of AML typically measure 5-6 cm [1], the tumor was notably larger in our case, measuring 9.3 x 8.2 x 7.5 cm. The most concerning complication is microaneurysm rupture, leading to retroperitoneal hemorrhage, as observed in our case, termed Wunderlich syndrome, which can be life-threatening [2,3,4]. Diagnosis relies on clinical examination, positive family history, and imaging modalities like CT, MRI, and ultrasound [2]. In our case, a distinct, hyperechoic lesion on ultrasound, coupled with fatty adhesions on CT, led to the diagnosis of renal AML (Table 2). Nonetheless, rare instances of the absence of fatty adhesions have been documented, posing challenges in distinguishing it from renal cell carcinoma [7,8].

**Table 2:** Correlation of clinicopathological features with other studies CT: computed tomography; USG: ultrasound sonography

No.	Study	Age (years)/Sex	Size (cm)	Clinical presentation	Imaging findings	Histopathology	Treatment
1	Chen et al. [4]	34/female	29	Progressive bloating sensation	USG - hyperechoic mass; CT - large mixed-density mass with fatty lobule	Smooth muscle, mature adipose cells, and thick-walled blood vessels	Left total nephrectomy
2	Alshehri et al. [5]	22/female	30	Right abdominal swelling, mild pain	CT - microscopic visible fat, multicystic fluid-filled mass; chest X-ray - normal	Smooth muscle with edematous stroma, fat lobules, and thickened blood vessels	Right radical nephrectomy
3	Lu et al. [7]	60/male	8	Asymptomatic (routine checkup)	CT - mixed hypodense and isodense cystic lesion with liquid density with no fatty lobules	Smooth muscle cells arranged in fascicles, mature adipose tissue, and partly hyalinized blood vessels	Left radical nephrectomy
4	Present Case	32/female	9	Recurrent bilateral flank pain	USG - bilaterally enlarged kidneys with multiple anechoic cysts and a hyperechoic lesion in the left kidney; CT - lobulated hyperdense lesion with fat attenuation	Smooth muscle cells arranged in a fascicular pattern, intermixed with adipose tissue, and blood vessels of varying thickness	Left radical nephrectomy

The treatment strategy for renal AML varies based on factors such as size, symptoms, and the presence of hemorrhages. Tumors larger than 4 cm, particularly if symptomatic, typically require surgical intervention, such as partial or radical nephrectomy [3,4], as was performed in our case. The patient reported no postoperative complaints, and a review ultrasound confirmed these findings. The patient reported no complaints During an eight-month follow-up.

## Conclusions

We presented a rare case of renal AML in a patient with co-existing TSC and associated polycystic kidney disease. The patient initially presented with bilateral flank pain, which, based on ultrasound and CT imaging, was confirmed to have been caused by a dual occurrence of ADPKD bilaterally and AML in the left kidney. We opted for left radical nephrectomy as the treatment of choice, after which the patient experienced no complications or complaints. Clinicians must consider AML as a potential differential diagnosis in patients with polycystic kidney disease or tuberous sclerosis and be knowledgeable about the available diagnostic and treatment modalities.
